# Impaired Hematopoietic Stem/Progenitor Cell Traffic and Multi-organ Damage in Diabetes

**DOI:** 10.1093/stmcls/sxac035

**Published:** 2022-05-12

**Authors:** Gian Paolo Fadini, Mattia Albiero

**Affiliations:** Department of Medicine, University of Padova, Padua, Italy; Veneto Institute of Molecular Medicine, Padua, Italy; Department of Medicine, University of Padova, Padua, Italy; Veneto Institute of Molecular Medicine, Padua, Italy

**Keywords:** adult hematopoietic stem cells, bone marrow, CD34+, diabetes, mobilization

## Abstract

During antenatal development, hematopoietic stem/progenitor cells (HSPCs) arise from a specialized endothelium and migrate from the extraembryonic mesoderm to the fetal liver before establishing hematopoiesis in the bone marrow (BM). It is still debated whether, in adulthood, HSPCs display such ontologic overlap with vascular cells and capacity for endothelial differentiation. Yet, adult HSPCs retain a prominent migratory activity and traffic in the bloodstream to secondary lymphoid organs and all peripheral tissues, before eventually returning to the BM. While patrolling parenchymatous organs, HSPCs locate close to the vasculature, where they establish local hematopoietic islands and contribute to tissue homeostasis by paracrine signals. Solid evidence shows that diabetes mellitus jeopardizes the traffic of HSPCs from BM to the circulation and peripheral tissues, a condition called “mobilopathy.” A reduction in the levels of circulating HSPCs is the most immediate and apparent consequence, which has been consistently observed in human diabetes, and is strongly associated with future risk for multi-organ damage, including micro- and macro-angiopathy. But the shortage of HSPCs in the blood is only the visible tip of the iceberg. Abnormal HSPC traffic results from a complex interplay among metabolism, innate immunity, and hematopoiesis. Notably, mobilopathy is mechanistically connected with diabetes-induced myelopoiesis. Impaired traffic of HSPCs and enhanced generation of pro-inflammatory cells synergize for tissue damage and impair the resolution of inflammation. We herein summarize the current evidence that diabetes affects HSPC traffic, which are the causes and consequences of such alteration, and how it contributes to the overall disease burden.

## Graphical Abstract

**Figure:**
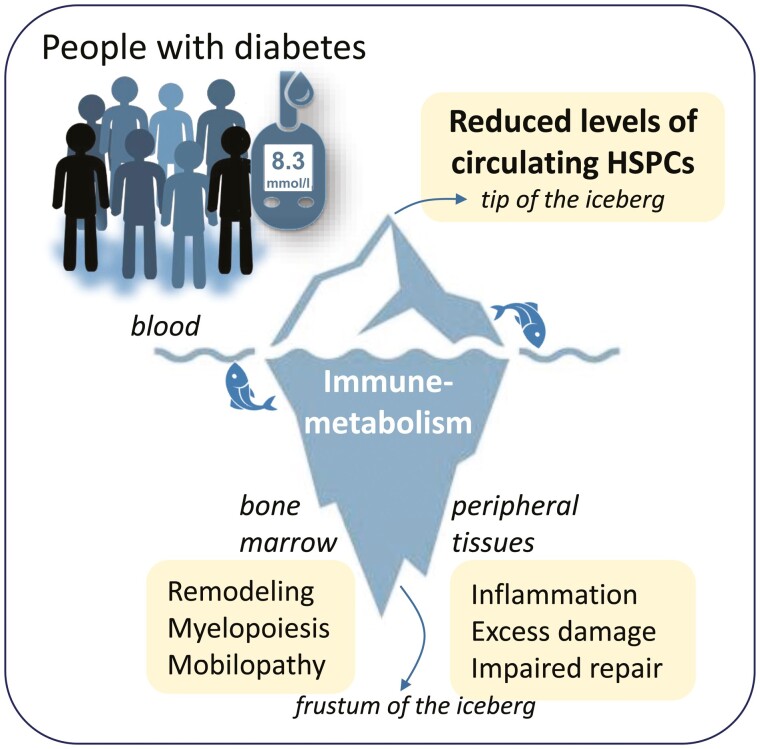

**Significance Statement**

Hematopoietic stem/progenitor cells (HSPCs) play key roles in maintaining hematopoiesis in the bone marrow but also migrate the circulation to help immune function and tissue homeostasis. Diabetes impairs such traffic of HSPCs. The resulting low level of HSPCs in the peripheral blood of individuals with diabetes is associated with several adverse health consequences including micro- and macrovascular disease and even worse COVID-19 prognosis. We herein unveil what alters HSPC traffic in diabetes and which are the consequences.

## Introduction to HSPC Traffic

The function of hematopoietic stem/progenitor cells (HSPCs) is to generate all mature blood cells, at the same time maintaining hematopoiesis throughout life. Self-renewal, asymmetric division, and differentiation are instrumental to the hematopoietic function, which takes place within the bone marrow (BM). A highly specialized BM microenvironment allows immature HSPCs to remain quiescent, while promoting the differentiation of committed HSPCs along the myeloid and lymphoid lineages.^1^ Despite HSPC exert most of their function within the BM, they retain a strong migratory capacity acquired during antenatal development and can reach the peripheral circulation, with a process called mobilization. The existence of circulating HSPCs, with an average level of 3 cells/µL, is known for 50 years,^2^ but the biological meaning of such a small cell population remained elusive for a decade and is still incompletely understood.

Far from representing a passive spillover from the BM, HSPC mobilization into peripheral blood (PB) is tightly regulated during physiologic and pathologic conditions. Migratory signals for HSPCs culminate in the modification of chemokine gradients between BM and PB, particularly for CXCL12 (alternatively known as stromal-derived factor-1 alpha [SDF-1α]). With high CXCL12 concentrations provided by BM stromal cells, HSPCs remain adherent to the BM stroma by virtue of several molecular interactions involving integrins, cadherins, selectins, and a variety of matrix proteins.^3,4^ Lack of CXCL12 signal and internalization of its receptor CXCR4 induces cellular modifications, such as nitric oxide production and activation of matrix metalloproteases, which loosen interactions with the stroma allowing HSPCs to follow higher CXCL12 concentrations in PB. These events are coordinated by local and systemic clues, including physical, hormonal, and neurological regulation. Of note, the release of HSPCs follows a circadian oscillation governed by a noradrenergic (sympathetic) and cholinergic (parasympathetic) interplay.^5-7^

The continuous recirculation of HSPCs in and out of the BM is instrumental to multifaceted functions that go beyond the steady-state production of blood cells. Trafficking through PB and back to the BM, HPSCs relocate to preferred niches that provide them with signals allowing maintenance or differentiation on demand.^8^ In fact, HSPCs that have left the BM to retain the ability to repopulate the BM, but display a myeloid-skewed profile.^9^ Furthermore, HSPCs patrol peripheral lymphoid tissues exerting immune-surveillance activity and seeding local hematopoiesis close to areas of inflammation. Therefore, thanks to their migratory activity, HSPCs can survey peripheral organs and can foster the local production of tissue-resident myeloid innate immune cells, especially in response to inflammatory signals, eg, via Toll-like receptor agonists.^10^

HSPCs can also enter parenchymatous organs where they contribute to local homeostasis. The vascular tropism of HSPCs is reminiscent of their origin from the hemogenic endothelium in the extraembryonic yolk sac mesoderm. Today, it is disputable whether HSPCs give rise to endothelial progenitor cells (EPCs) fully able to generate mature endothelial cells constitutively populating the vasculature. Even if the capacity for this retro-differentiation to the embryonic hemangioblast is likely lost in adulthood, HSPCs still have a strong preference for locating very close to blood vessels in the BM as well as in other tissues. For example, human CD34^+^ HSPCs were transplanted into pre-gastrulation zebrafish co-localized with the putative hemangioblasts, characterized by Scl and Gata2 expression. When transplanted into post-gastrulation zebrafish, human HSPCs were recruited into developing vessels, exerted paracrine activities, and enhanced vascular repair.^11^

Based on these premises, it is easy to anticipate how alterations in the traffic of HSPCs can have profoundly adverse consequences for homeostasis as a whole. In view of the role exerted by the BM environment in regulating HSPCs survival and mobilization, this highly specialized tissue is being recognized as a central housekeeper of global organismal health.

Here, we review the available evidence on alterations of HSPC traffic in diabetes and how this can impact the development and progression of multi-organ damage of chronic diabetic complications.

## Method for Conducting This Review

We searched the medical literature published in English up to March 2022. We first run the following string on PubMed: (“hematopoietic”) and (“stem” or “progenitor”) and (“traffic” or “mobilization” or “migration”) and (“diabetes” or “diabetic”). We retrieved and manually screened 212 records. We then excluded articles not dealing with diabetes or with HSPC traffic/mobilization and selected those providing the most robust information in terms of mechanistic insight or clinical implications. Other articles were selected by notoriety in the field and by screening the reference list of the retrieved publications.

## Relevance of HSPCs in Diabetes

Due to the widespread of obesity and unhealthy lifestyle, type 2 diabetes (T2D) is a growing concern for healthcare systems worldwide. The global diabetes prevalence in 2019 was estimated to be 9.3% (463 million people), rising to 10.2% (578 million) by 2030 and 10.9% (700 million) by 2045.^12^ Diabetes leads to an increase in the risk of multi-organ damage that drives chronic complications. In addition to typical sites where traditional complications occur (retinopathy, nephropathy, neuropathy, and cardiovascular disease), diabetes can affect the structure and/or functioning of most tissues in the human body, also including the skin, liver, lungs, bones, and bowel. The relevance of T2D diffusion was appreciated also during the COVID-19 pandemic, as T2D represents a major risk factor for severe COVID-19 requiring intensive care and eventually leading to death.^13^ Type 1 diabetes (T1D), although >10 times rarer than T2D, shares the same wide spectrum of multi-organ damage and, from a research perspective, represents an ideal benchmark to study the adverse effects of hyperglycemia without the confounding of concomitant risk factors that accompany T2D (obesity, hypertension, and dyslipidemia). Owing to the extensive complication burden, diabetes at the age of 50 shortens life expectancy by approximately 6-7 years in both men and women.^14^ Cardiovascular diseases are responsible for ~60% of excess mortality in people with diabetes,^15^ but diabetes also increases the rate of death from cancer, liver diseases and non-hepatic digestive diseases, renal disease, pneumonia and other infectious diseases, chronic obstructive pulmonary disease, mental disorders and nervous-system disorders, and even external causes including intentional self-harm.^14^ The exact causes driving such excess morbidity and mortality are not entirely known. The most obvious interpretation is that, in addition to increasing cell and tissue damage through the toxic effect of hyperglycemia and the associated dysmetabolic milieu, diabetes compromises the physiological processes of tissue repair.^16^ Thus, it is conceptually easy to find similarities between diabetes and accelerated aging syndromes, such as those due to impaired mismatch repair.^17^ Notably, such aging syndromes (eg, Fanconi anemia) feature BM failure and decline in the functioning of HSPCs.^18^

**Figure 1.**
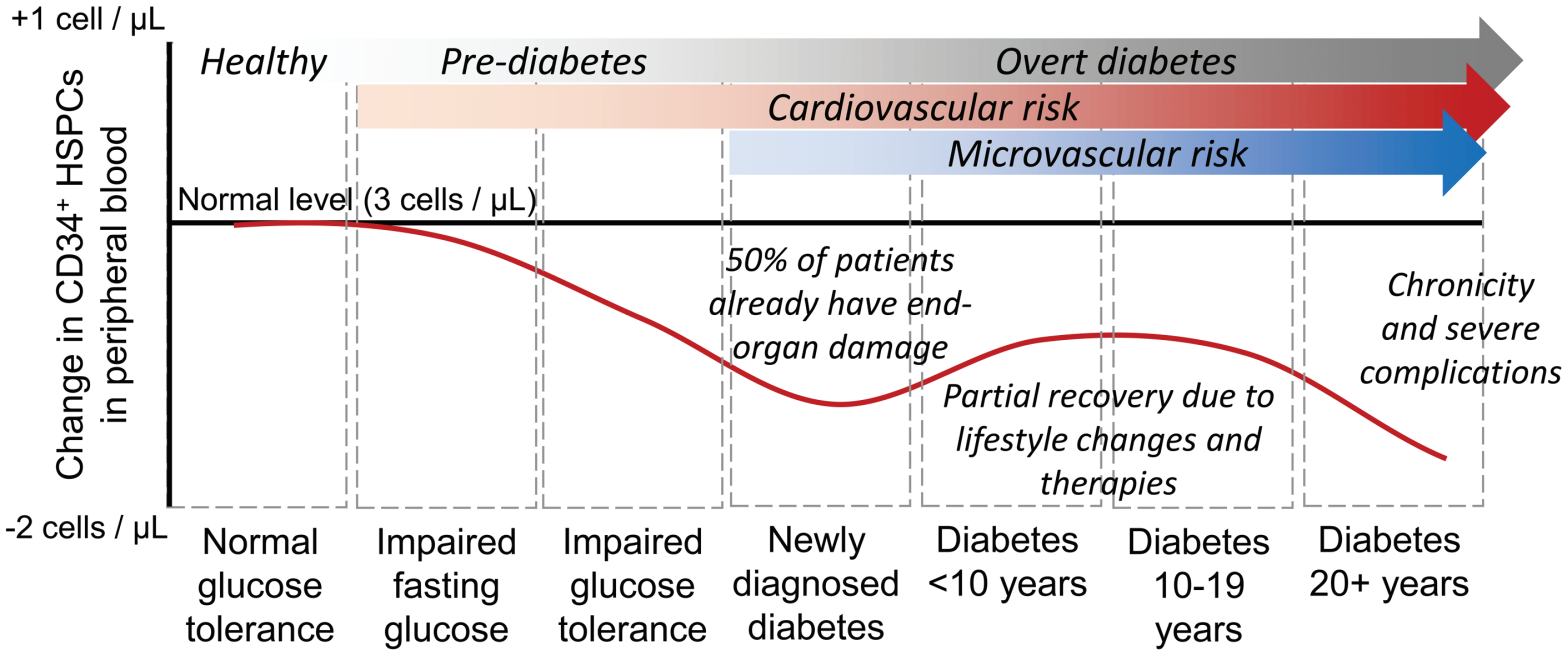
Circulating HSPC during the natural history of type 2 diabetes. Modified from Fadini et al.^19^.

In 2010, we observed that the levels of circulating CD34^+^ HSPCs were significantly reduced by 30-40% in people with T2D, as compared to those without, and mirrored the disease’s natural history.^19^ Shortage of circulating HSPCs was confirmed by several investigators^20^ and is also observed in T1D.^21^ We described an early decline in pre-diabetic individuals and the first nadir in HSPC levels in newly-diagnosed T2D patients. This probably reflects a status of enhanced susceptibility to damage in the early stages of T2D and is paralleled by the common observation that up to 50% of patients with newly diagnosed T2D have clinical or preclinical manifestations of microvascular and/or macrovascular complications.^22^ Then, we observed that circulating HSPC tended to return toward levels observed in control individuals, possibly as a result of lifestyle intervention and pharmacological treatments, before finally dipping again 20 years or more from diagnosis.^19^ Indeed, physical exercise and diabetes pharmacotherapies have the potential to increase the levels of HSPCs in people with diabetes,^23-25^ but the long-term persistence of such effect is unknown (Fig. 1).

Several researchers embarked on pre-clinical studies to examine the causes and consequences of the consistently reduced levels of circulating HSPCs in diabetes.

## HSPC Traffic in Diabetes

**Figure 2.**
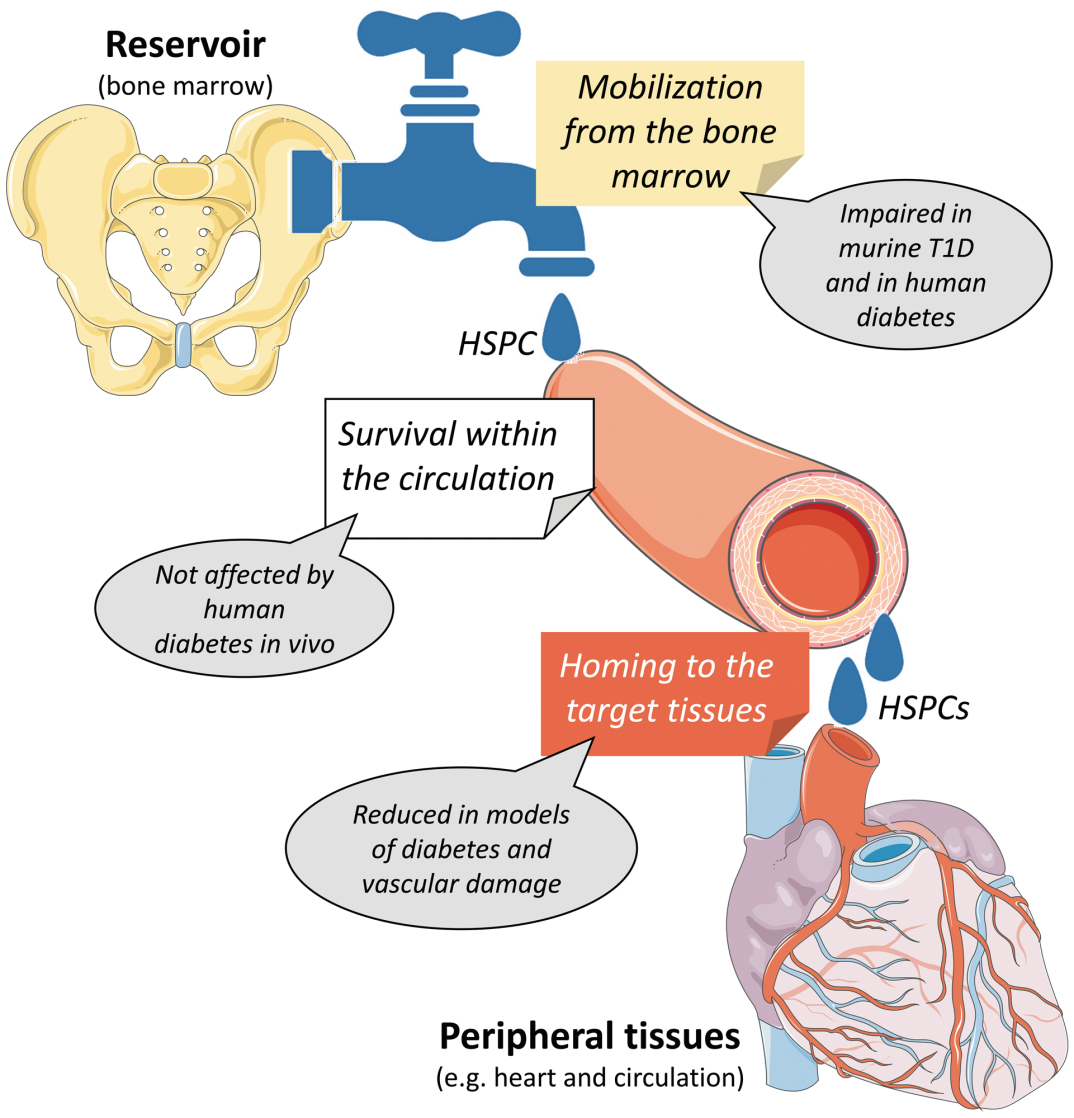
Three-compartment model of HSPC traffic. According to a simplistic 3-compartment model, changes in the levels of circulating HSPCs can derived from alterations in replenishment from the bone marrow reservoir, reduced survival in the bloodstream, and excess homing to the target tissues. T1D, type 1 diabetes.

In a simplistic 3-compartment model, a shortage of HSPCs in PB may result from (i) defective replenishment from the BM; (ii) shortened survival in PB; (iii) enhanced migration from PB to target tissues (Fig. 2). In vitro, high glucose increased the apoptosis of CD34^+^CD45^-^ progenitor cells.^26^ However, in vivo, the apoptotic rate of CD34^+^ HSPCs in PB in patients with T2D was similar to that of non-diabetic counterparts and HSPC apoptosis was explaining only 10-12% of the variation in their circulating levels.^19^ Therefore, cell death is unlikely to account for the profound reduction in HSPCs observed in diabetic PB. One could argue that HSPCs more often leave PB because diabetes increases tissue damage and demand for tissue repair. We found no evidence that this occurs in vivo. In a murine model of wound healing, recruitment of BM-derived cells was impaired by experimental T1D-like streptozotocin (STZ)-induced diabetes.^27^ Similarly, recruitment of BM-derived cells to areas of carotid endothelial damage, including murine HSPCs (LKS cells, Lin^-^Sca1^+^c-Kit^+^), was impaired in STZ diabetes.^28^ These data provide no support to the hypothesis that HSPCs are “wasted” in an attempt to repair ongoing tissue damage. Rather, the traffic of HSPC to the site of tissue damage is impaired by diabetes, thereby contributing to the defect in tissue repair.

Then, studies concentrated on the hypothesis that shortage of HSPCs is mainly driven by a defect in mobilization from the BM. In 2006, while studying the response to remote tissue ischemia in STZ diabetic rats, we first demonstrated an impairment in the release of BM progenitor cells. Of note, this occurred both in response to hind limb ischemia and after direct BM stimulation with G-CSF and SCF.^29^ Defective BM-progenitor cell release was observed in a murine model even after myocardial ischemia,^30^ highlighting a generalized derangement of ischemic responses. The impaired BM-HSPC mobilization response after G-CSF was confirmed by other groups in the mouse T1D model^31^ and then confirmed in human diabetes. In a prospective trial, we found that low-dose (5 μg/kg) human recombinant G-CSF was able to double the levels of circulating HSPCs in healthy controls after 24 h (from ~3 to ~6 cells/μL), but exerted no effect in people with T1D or T2D, whose levels of HSPCs remained unchanged.^32^ Such findings in humans have been supported by 2 retrospective studies on a small (*n* = 62)^31^ and a large (*n* = 1041)^33^ cohort of patients with cancer undergoing G-CSF stimulation for autologous or allogeneic PB-HSPC transplantation. Such solid evidence of impaired HSPC mobilization in diabetes granted the new definition of “diabetic stem cell mobilopathy”^34^ to describe a previously neglected form of complication. The “poor mobilizer” condition associated with diabetes has implications for the care of patients receiving PB-HSPC transplantation,^35^ but has ramifications that impact the risk for future micro- and macro-vascular complications (see below).

## Molecular Mechanisms of the Diabetic Stem Cell Mobilopathy

It is important to note that the mobilization impairment was observed in both forms of diabetes and irrespectively of disease duration, glycaemic control, and of presence/absence of overt complications. This seems to suggest that hyperglycemia and disease chronicity could exert different and independent detrimental effects on BM function. Hyperglycemia likely activates molecular pathways that functionally prevent HSPC mobilization, whereas later end-organ damage compromises BM structure and function irreversibly. Indeed, remodeling of the BM stem cell niche has been shown in experimental diabetes models, featuring microangiopathy, damage to nerve fibers, and extensive marrow fat infiltration.^36-39^ Due to the very specialized function of BM niche components,^1^ all these anatomical alterations can dramatically impact HSPC traffic. Since the mobilizing activity of G-CSF requires a functional intra-marrow sympathetic nervous system,^7^ autonomic neuropathy occurring in human and experimental diabetes is a primary contributor to stem cell mobilopathy and to the consequent impairment in vascular recovery after ischemia.^39^ High-dose G-CSF used to induce mobilization also activates sensory nerve fibers in the BM and cholinergic signals are also been implicated in the HSPC traffic.^5,40^ Therefore, several manifestations of diabetic neuropathy, involving the sympathetic, parasympathetic and sensory components, can blunt HSPC mobilization. As a clinical counterpart, bone pain, which is caused by G-CSF acting on the BM and correlates with the extent of mobilization, is alleviated by diabetes and is associated with an impaired HSPC release.^38^ Therapeutically, mobilopathy due to BM neuropathy could be overcome by deleting the redox enzyme p66Shc that drives oxidative stress nerve damage, by overexpressing the longevity protein Sirt-1, by boosting the residual nerve function with the norepinephrine reuptake inhibitor desipramine,^39^ or by stimulating nerve regeneration with nerve growth factor.^41^

The highly specialized BM vasculature is another key player in the regulation of HSPC egress.^42^ Both murine and human diabetes exhibit rarefaction of the capillary network and distortion of sinusoids.^36,43,44^ As in other tissues, diabetes increases vascular permeability in the BM, which is instrumental for the traffic of HSPCs.^42^ Even in the absence of anatomical microangiopathy, BM endothelial barrier dysfunction in murine T1D is at least in part attributable to oxidative stress-mediated activation of Rho kinases,^45^ one of the functional consequences of hyperglycemia that are reversible upon restoration of glycaemic control with insulin therapy. Experimental diabetes induces a change in the transcriptomic profile of BM endothelial cells involving Cxcl12 and genes of the Egfr signaling, which regulate HSPC retention and quiescence.^46^ BM adipocytes are an important source of Cxcl12 that retain HSPCs within the BM niche, preventing them from being mobilized into PB.^37^ Cxcl12 is produced by several stromal cell types within the BM niche and signals in HSPCs via its receptor CXCR4. There is solid evidence to support that the diabetic stem cell mobilopathy relies on the downstream retaining signal of Cxcl12 in HSPCs. Mobilopathy is associated with impaired regulation of the Cxcl12-cleaving enzyme CD26/DPP-4^32^ and activity of DPP-4 is associated with alterations in the compartmentalized traffic of HSPCs.^47^ Most importantly, inhibiting Cxcl12 signal by the CXCR4 antagonist plerixafor allows rapid and effective HSPC mobilization in mice and humans with diabetes as it does in the non-diabetic counterpart.^31,33^ We have found that BM stromal cells are induced to express and release Cxcl12 in response to the IL-6 cytokine member Oncostatin M (OSM).^48^ One of the major sources of OSM within the BM are macrophages with a pro-inflammatory (so-called M1) phenotype and identified by surface expression of the sialoadhesin CD169 (SIGLEC-1) in both mice and humans.^48^ It was previously found that selective depletion of BM CD169^+^ macrophages with targeted expression of diphtheria toxin synergized with G-CSF to induce HSPC mobilization into PB.^49^ Reprogramming of BM macrophages toward an anti-inflammatory phenotype using a PPAR-γ agonist is another strategy to prevent OSM overexpression and counter mobilopathy in humans and mice.^37^ CD169^+^ macrophages are now considered as an integrated component of the BM niche, regulating HSPC traffic and differentiation, as well as erythropoiesis.^50,51^ The mechanisms whereby diabetes increases the BM content of pro-inflammatory macrophages are probably manifold. Hyperglycemia in experimental T1D, by promoting the release of alarmins S100A8/A9 fosters the differentiation of BM-HSPCs along the myeloid lineage, giving rise to neutrophils and monocytes primed to M1 macrophage differentiation.^52^ In the setting of obesity and models of T2D, alarmins from the adipose tissue activate the inflammasome in the BM driving myelopoiesis.^53^ In both cases, excess myelopoiesis increases the ratio of neutrophil to lymphocyte (N/L) count in PB, a well-known marker of cardiovascular risk.^54,55^ The same pathways appear to be responsible for abnormal activation of thrombopoiesis,^56^ a substantial contributor to cardiovascular disease in diabetes.^56^ Therefore, pro-inflammatory myeloid cells propagate to the vasculature favoring the progression of atherosclerotic plaques, where increased and activated platelets would promote thrombosis and vascular occlusion. As a clinical counterpart of this enhanced myelopoiesis, recent data illustrate how the BM is metabolically over-activated, as evidenced by 18F-FDG uptake, in patients with the metabolic syndrome and early signs of atherosclerosis.^57^ On the other side, as a result of the same myelopoietic stimulus, excess pro-inflammatory macrophages residing in the BM increases the production and signaling of OSM, retaining HSPC in the niche and nourishing a vicious cycle wherein more HSPCs undergo myeloid differentiation that sustain systemic inflammation. Recently, it has been shown that activated neutrophils, themselves a source of OSM,^58^ can home back to the BM where they sustain granulopoiesis and worsens myocardial function after experimental ischemia.^59^

**Figure 3.**
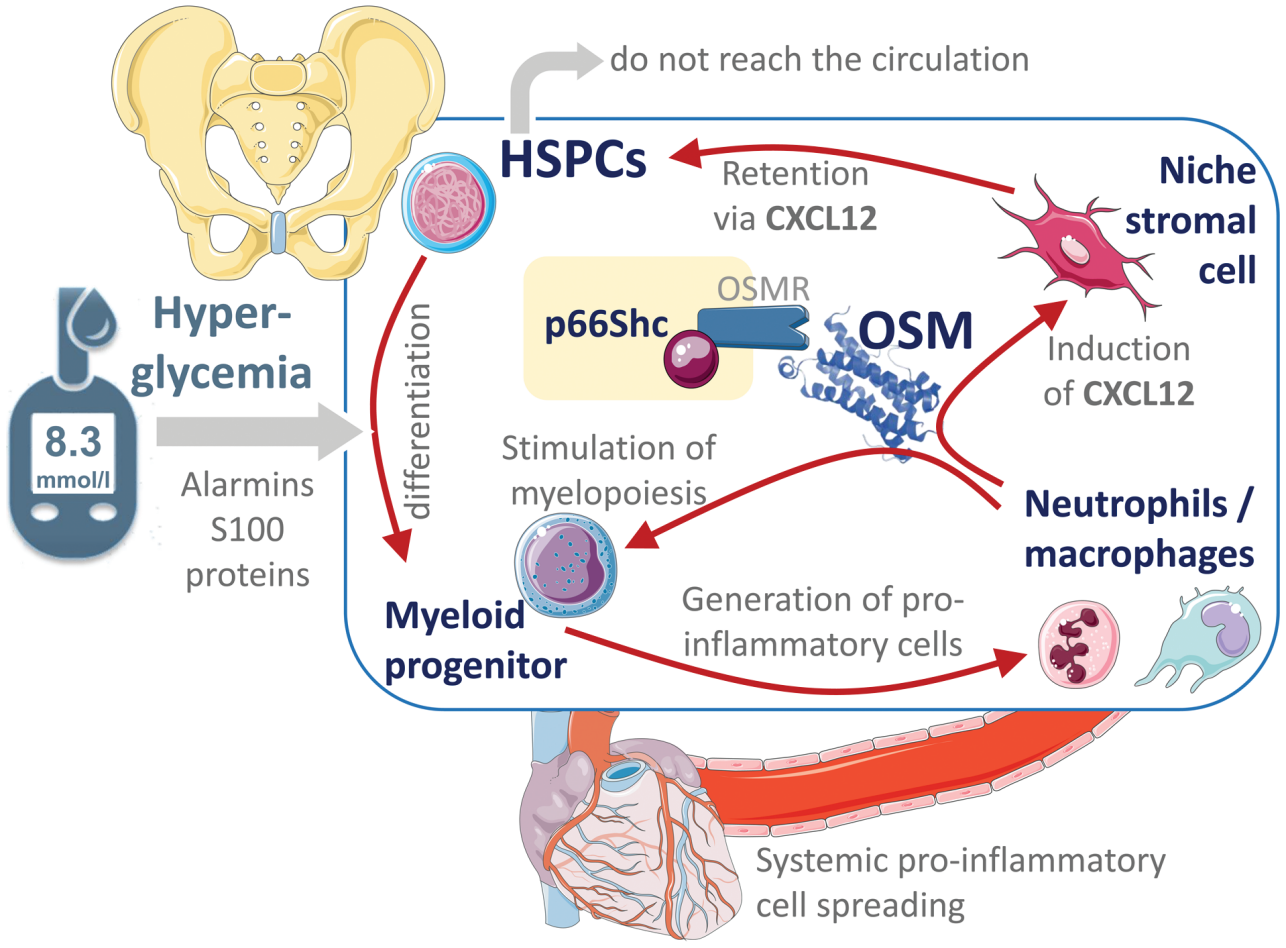
Mechanistic link between myelopoiesis and mobilopathy in diabetes. The figure reads from left to right and in a circular manner. Hyperglycemia in people with diabetes, possibly through the release of alarmins (like S100A8/9 proteins) from activated granulocytes, promotes the myeloid biased differentiation of hematopoietic stem/progenitor cells (HSPCs), resulting in an exceeding amount of myeloid progenitors in the diabetic bone marrow. Myelopoiesis leads to an increased release of pro-inflammatory neutrophils and macrophages that spread through the bloodstream and reach targets organs of diabetic complications like the heart. Within the bone marrow, pro-inflammatory cells produce Oncostatin M (OSM), which signals through its receptor (OSMR) via non-mitochondrial p66Shc to induce CXCL12 by niche stromal cells and itself alimenting myelopoiesis. Local production of CXCL12 retains HSPCs in the niche, preventing them from being mobilized, and nourishing the vicious cycle of the myelopoiesis-mobilopathy combo.

OSM in the BM exerts paracrine and autocrine functions. Transcellular OSM signaling in stromal cells culminates in Cxcl12 transcription and secretion and is mediated by non-mitochondrial p66Shc recruitment to the plasma membrane as a docking for the intracellular transduction. Intrinsically within the hematopoietic cell compartment, OSM itself sustains myelopoiesis and p66Shc is also required for S100A8/A9 to drive HSPCs along the myeloid lineage.^60^ Deletion of either OSM or downstream p66Shc was in fact able to lower the N/L ratio and to rescue HSPC mobilization.^60^ Furthermore, in a mouse model of T1D, selective deletion of p66Shc in the hematopoietic system, by disarming the anti-mobilizing and myelogenous effect of OSM, was sufficient to restore HSPC traffic to sites of ischemia and improve blood flow recovery.^61^Fig. 3 illustrates the central role of OSM and p66Shc in the link between myelopoiesis and mobilopathy.

## Contribution to Multi-Organ Damage

Diabetes compromises the structure and function of any microvascular tissue in the human body, accounting for the traditional complications (retinopathy, nephropathy, and neuropathy), but also contributing to excess morbidity and mortality in several other diseases due, for example, to lung, liver, and skin microangiopathies. Vascular tropism of HSPCs, which relocate to branching sites and sprouts of the damaged microcirculation,^62^ explains why alterations in their traffic can worsen microangiopathy and compromise tissue homeostasis. To demonstrate whether patients with low circulating HSPCs are in fact exposed to a higher complication burden over time, we enrolled a cohort of 187 patients with T2D, who were extensively characterized and followed over time with gold-standard methodologies to assess end-organ damage. After 3.9 years, below-median levels of circulating CD34^+^ HSPCs were associated with new-onset diabetic retinopathy, nephropathy, and neuropathy, or worsening of these preexisting microangiopathies, independently from typical confounders (age, sex, diabetes duration, and glycaemic control) that drive the risk of complications.^63^

In parallel to microvascular damage, diabetic patients face a striking increase in cardiovascular risk, which has multifaceted mechanisms. Hyperglycemia causes endothelial cell dysfunction, priming the onset of atherosclerosis. The co-existence of other risk factors contributes to cardiovascular risk, with hyperglycemia and insulin-resistance acting as accelerators. Chronic low-grade inflammation plays a major role in favoring cardiovascular disease in people with T2D, although the exact mechanisms triggering the inflammatory response are unclear.^64,65^ In the same aforementioned cohort, after a median observation of 6.1 years, patients with low HSPCs displayed a 2-fold increased rate of major adverse cardiovascular events (cardiovascular death, nonfatal myocardial infarction, or nonfatal stroke) or hospitalization for cardiovascular causes than those in the higher cell level group.^66^ The adverse prognostic meaning of low HSPC levels with regards to cardiovascular disease has been extensively confirmed in other populations of patients, beyond diabetes.^67,68^ The combination of mobilopathy and myelopoiesis, rather than reduced HSPC alone, is likely responsible for such a strong association with poor patients’ outcomes. For example, it has been shown that the increase in IL-6 concentrations explains 40% of the higher cardiovascular event rate among people with low HSPCs.^69^

It is of great interest to underline how the prognostic impact of reduced circulating HSPCs are not limited to diabetic micro- and macroangiopathy. In the setting of COVID-19, diabetes and hyperglycemia have emerged worldwide as risk factors for poor outcomes, including admittance to the intensive care unit and death.^13,70^ Knowing that hyperglycemia is causally impairing HSPC mobilization, we explored whether reduced HSPCs mediated at least part of the prognostic effect of hyperglycemia on COVID-19 outcome. Even among COVID-19 patients, glucose concentrations were inversely correlated with HSPC levels and lower HSPCs were associated with a more than threefold higher risk of adverse outcomes in the short-and long term.^71^ Such finding was independent of confounders, including the N/L ratio, a well-known feature of hyper-inflammation in severe COVID-19.^72^

## Outstanding Questions and Concluding Remarks

A few outstanding issues remain concerning the regulation of HSPC traffic in diabetes. First, while mobilopathy is an extremely consistent finding in models of T1D, models of T2D can display heterogeneous mobilization in response to G-CSF. For example, leptin receptor (Lepr)-deficient db/db mice seem to have an enhanced release of HSPCs,^73^ which may be related to the role played by Lepr in BM niche stromal cells as well as in HSPC themselves.^74,75^ However, also in the murine model of T2D and obesity induced by high-fat diet, mobilization was not defective but rather enhanced.^28^ Interestingly, some groups have reported that, in models resembling conditions of high atherosclerotic risk, HSPCs and multipotent progenitor cells are forced to leave the BM and establish extra-medullary hematopoiesis in the spleen.^52,76-78^ While this enhanced traffic observed in animal models is not normally seen in patients with T2D or the metabolic syndrome,^79^ it may occur in young individuals during the dynamic phase of adipose tissue expansion, fostering the development of dysmetabolic consequences.^80^ Among initially healthy individuals aged 46 years, we found that circulating CD34^+^ HSPCs were higher in obese ones who subsequently increased their adiposity indexes and developed features of the metabolic syndrome over the following years.^80^ This clinical condition is what may most resemble mice during their dynamic phase of weight gain, but this very specific example reminds us that human disease may not always reflect what is seen in murine models.

Finally, it remains to be elucidated if and to what extent, restoring HSPC traffic improves tissue repair in human diabetes. We have shown that effectively mobilizing HSPCs from the BM to PB without countering myelopoiesis did not improve wound healing in patients with diabetes and critical limb ischemia (CLI).^81^ Consistently, Spinetti et al. found that excess migratory activity of CD34^+^ HSPCs was associated with an increased risk of cardiovascular events among patients with diabetes and CLI, possibly because HSPCs from those patients turned into anti-angiogenic and pro-apoptotic elements.^82^ Similarly, we speculate that countering mobilopathy and myelopoiesis at the same time is needed to promote tissue repair because enhancing the traffic HSPCs with a strong tendency to myeloid differentiation may even worsen tissue damage and clinical outcomes. Notably, selective inhibition of p66Shc in the BM would antagonize both mobilopathy and myelopoiesis and rescue response to ischemia.^61^ A similar effect may be obtained with SGLT-2 inhibitors, which are provided with extensive cardio-renal benefits in patients with T2D.^83^ For example, treatment of diabetic mice with dapagliflozin was able to restrain BM myelopoiesis, partly restore HSPC mobilization, rescue homing of HSPC to the injured endothelium, and finally improve endothelial repair by the contribution of circulating cells.^28^ Whether the observed clinical benefits of SGLT-2 inhibitors truly rely on the improvement in HSPC traffic is unknown and deserves further investigation.

The clinical significance of a sole reduction of PB-HSPCs from ~3 to ~2 cells/µL was long questioned^84^ and attributing strong biological and clinical consequences to this observation remains challenging.

The current mechanistic understanding that the shortage of circulating HSPCs is a reflection of altered HSPC traffic and results from a series of pathologic events that involve the BM and the hematopoietic system provides a more comprehensive view of the link with diabetic end-organ damage. The next challenge will be translating such concepts into new therapeutics against diabetic complications.

## Data Availability

No new data were generated or analyzed in support of this research.
